# Go and stop signals for glial regeneration

**DOI:** 10.1016/j.conb.2017.10.011

**Published:** 2017-12

**Authors:** Alicia Hidalgo, Ann Logan

**Affiliations:** 1 School of Biosciences, University of Birmingham, UK; 2 Neuroscience and Ophthalmology, College of Medicine and Dentistry, University of Birmingham, UK

## Abstract

The regenerative response of ensheating glia to central nervous system (CNS) injury involves proliferation and differentiation, axonal re-enwrapment and some recovery of behaviour. Understanding this limited response could enable the enhancement of it. In *Drosophila*, the glial progenitor state is maintained by Notch, an activator of cell division and Prospero (Pros), a repressor. Injury provokes the activation of NFκB and up-regulation of Kon-tiki (Kon), driving cell proliferation. Homeostatic switch-off comes about as two negative feedback loops involving Pros terminate the response. Importantly, the functions of the *kon* and *pros* homologues *NG2* and *prox1*, respectively, are conserved in mammalian NG2 glia. Controlling these genes is key for therapeutic manipulation of progenitors and stem cells to promote regeneration of the damaged CNS.

## Highlights

• The glial regenerative response to CNS injury is evolutionarily conserved.
• Glial proliferation and differentiation critically depend on the genes *kon-tiki/NG2* and *pros/prox-1*.
• Two positive feedback loops provide ‘go-signals’ that induce a rapid regenerative response.
• Two negative feedback loops deliver ‘stop signals’ that terminate the response.
• Injury re-activates a developmental mechanism that normally maintains structural homeostasis.

**Current Opinion in Neurobiology** 2017, **47**:182–187

This review comes from a themed issue on **Glial biology**

Edited by **Alison Lloyd** and **Beth Stevens**

For a complete overview see the Issue and the Editorial

Available online 7th November 2017

**http://dx.doi.org/10.1016/j.conb.2017.10.011**

0959-4388/Crown Copyright © 2017 Published by Elsevier Ltd. This is an open access article under the CC BY license (http://creativecommons.org/licenses/by/4.0/).

Regeneration occurs in some animals, revealing that in principle cells might ‘know’ how to achieve and restore organismal integrity. However, the adult mammalian and insect central nervous system (CNS) do not regenerate upon damage, disease or injury. This leads to permanent disability, and an important neuroscience goal is to discover how to enhance CNS regeneration. Both in mammals and fruit-flies, injury induces a stereotypic response that reveals a natural yet limited tendency of the CNS to mend itself: the lesion first expands and then shrinks [1, 2••]. Lesion expansion correlates with increased cell death and the formation of vacuoles; whilst shrinkage correlates with the activation of glial repair and regenerative responses [1, 2••, 3]. ‘Repair’ means to restore something damaged to a good condition; ‘regenerate’ means to grow again. Different glial cell types elicit distinct responses [1].

Some glial cell types repair the damaged site, by clearing cell debris and forming a glial scar. Debris clearance is initiated by an inflammatory reaction, glial cells migrate to the lesion, engulf and dissolve axonal fragments, apoptotic cells and vacuoles [1]. In the adult mammalian CNS, this response is carried out by microglia (supported by extravasated monocytes), and most glial cell types can become phagocytic in *Drosophila* [2••, 4, 5]. The rapid formation of a glial scar isolates the wound, restores tissue barriers and prevents further tissue expansion. It is elicited by activated astrocytes in mammals. This response may have evolved in warm-blooded animals, as no scar appears to form upon injury in the fish or insect CNS [2••, 6, 7••, 8]. These glial repair responses will not be dealt with further here.

Glial cells of ensheathing cell lineages regenerate themselves upon injury [1]. Ensheathing glial cell lineages, like oligodendrocyte progenitor cells (OPCs, also called NG2 glia) in mammals respond to injury by undergoing compensatory proliferation to regenerate themselves, provide trophic support for neurons and re-enwrap axons, leading to some recovery of neuronal function and behaviour [1, 8, 9, 10, 11•]. This regenerative response is evolutionarily conserved across animal phyla, for example, in insects, fish, rodents and humans. In the cockroach, injury induces extensive glial proliferation followed by recovery of normal conduction [12]. In *Drosophila*, neuronal genetic ablation in the embryo, and stabbing and crush injury in the larva, all induce proliferation of axon-associated neuropile glia [2••, 7••, 13, 14•, 15•]. In fish, rodents and humans, injury induces oligodendrocyte (OL) death, followed by the regenerative response of NG2 glia described above, leading to remyelination [8, 11•]. This response is induced after spinal cord injury, traumatic brain injury and stroke, and correlates with the remitting phases of multiple sclerosis [1, 9, 16]. However, despite extensive NG2-glia proliferation after injury, insufficient daughter cells differentiate into OLs, limiting axonal re-enwrapment and functional recovery. A crucial challenge to regenerative biologists focused on functional rescue of the damaged mammalian CNS is to find out how to enhance the differentiation of OPCs into OLs, and their subsequent progression to remyelination [1, 17]. On the other hand, transplantation of olfactory ensheathing glia (OEG), NG2 glia and/or stem cells to the injury site, in the retina and spinal cord, have yielded encouraging results in the pursuit of functional restoration of the damaged CNS, in animal models and in humans [18, 19]. Why such remarkable functional recovery takes place is not understood, but as well as involving glial regeneration and remyelination, it is likely to involve also neuronal events. For instance, transplanted glia might provide trophic support for neurons, aid axonal navigation and re-growth. Either way, therapeutic cell transplantations rely on the prior precise manipulation of stem cells, OEG or OPCs [20]. In this context, discovering genes that control glial responses to injury, and their operating principles, is critical.

## Go and stops signals drive the glial regenerative response to CNS injury

**Figure 1:**
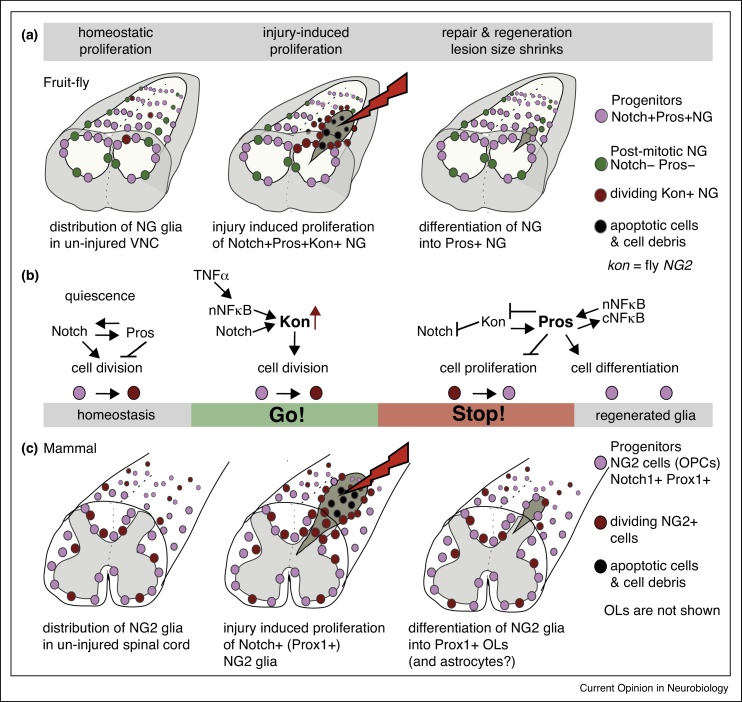
The glial regenerative response to CNS injury in fruit-flies and mammals. **(a)** The *Drosophila* larval ventral nerve cord. The Notch+Pros+NG (mauve) have cell bodies surrounding the neuropile, with part of their cytoplasms extending into regions of the neuropile, where axons and dendrites are located (white). Neuronal cell bodies and other glial cell types are located in the cortex (grey). Notch+Pros+NG divide during axon guidance, but they rarely divide in larval life, which lasts for five days. Divisions in larva are homeostatic. Upon injury to the larval VNC, the lesion first expands as many cells die. Injury induces compensatory proliferation of surviving NG. Subsequently, proliferation ceases, glial cells differentiate and the lesion shrinks. **(b)** Molecular mechanism underlying the glial regenerative response in *Drosophila.* NG are kept ‘ready to divide’ through the mutually dependent, yet antagonistic functions of Notch and Pros. Injury induces the activation of NFκB/Dorsal and Notch-dependent up-regulation of *kon* (homologue of *NG2*) expression. Kon induces proliferation of Notch+Pros+NG. Kon also activates the expression of *pros*. Pros inhibits proliferation and activates glial differentiation. Negative feedback by Kon on Notch, and by Pros on *kon* expression, terminates the response to injury. Pros regulates the expression of *NFκB/dorsal*, which remains in the cytoplasm ready to respond to future injuries. **(c)** The mammalian dorsal spinal cord. NG2 glia populate the white matter, that is, neuropile with myelinated axons (in white). Some of these NG2 glia normally divide producing meylinating oligodendrocytes (OLs, not shown). Injury induces cell death, lesion expansion and subsequent compensatory proliferation of remaining NG2 glia. Newly produced glia can then differentiate into astrocytes or OLs, and can spontaneously re-myelinate axons, as the lesion shrinks. Genes and resulting molecular mechanisms in these responses are evolutionarily conserved.

The fruit-fly *Drosophila* is a very powerful model organism to discover evolutionarily conserved molecular mechanisms. The neuropile of the *Drosophila* ventral nerve cord (VNC), which is equivalent to the mammalian dorsal spinal cord, is populated by neuropile glia (NG) [2••, 13]. NG have been subdivided into ‘astrocytes’ and ‘ensheathing glia’ [4, 21, 22, 23] (Figure 1a, mauve and green, respectively), but this nomenclature is not always helpful. The so called ‘astrocytes’ project into the neuropile and interact with synapses, but they also enwrap individual axons and clusters of axons. Larger axons are enwrapped individually, and thinner axons are enwrapped in clusters resembling Remak bundles of the mammalian peripheral nervous system [2^••^]. Axonal enwrapment is not as tight as in mammals, myelin is not produced, and no Nodes of Ranvier are formed [2^••^]. These glia express the genes *Notch* and *prospero* (*pros*) [2••, 13, 22, 24]. Pros is required for axonal enwrapment within the neuropile [2^••^]. In mammals, the *pros* homologue *prox1* is expressed in OPCs and OLs, but not astrocytes [25•, 26•]. The so-called ‘ensheathing glia’ do not enter the neuropile, but they wrap around the outside of the neuropile instead, cannot divide and do not express *Notch* or *pros* [2••, 4, 7••, 13, 21, 22]. Both Pros+ and Pros-negative glia express factors involved in neurotransmitter recycling [2••, 22], a feature shared with astrocytes, OPCs and OLs. Notch+Pros+NG are the only glia to retain mitotic potential and divide in development or upon injury [2••, 7••]. Like mammalian NG2 glia and Schwann cells, Notch+Pros+NG are at once progenitors and differentiated cells. Thus, NG share properties with mammalian astrocytes, NG2 glia/OPCs and OLs. To avoid further confusion, here we will refer to these cells called astrocytes by others, as Pros+NG.

Pros+NG proliferation is sensitive to neuronal interactions. During development, Pros+NG initially divide without G phases, and the G1 phase starts when NG contact axons bearing Serrate and Delta, which activate Notch signaling in these glia [13, 27, 28]. After axonal engagement, Notch+Pros+NG divide once more, and as glial cells are produced they sort axons into fascicles [13, 27]. After axon guidance, VNC NG do not divide further, and Notch+Pros+NG remain quiescent or slow cycling until at least the end of larval life (they have not been studied later) [2••, 13]. Notch+Pros+NG remain in a progenitor state as they retain mitotic potential and divide quickly if provided with Cyclin E [2••, 13]. In contrast, Notch-negative, Pros-negative NG cannot divide, even if provided with Cyclin E [13]. Mitotic potential is maintained by the combined action of Notch, an activator of cell division, and Pros, an inhibitor of cell division. *Notch* and *pros* maintain each other's expression, thus their antagonistic functions prevent cell division but keep cells ready to divide [13] (Figure 1b). Although divisions are rare, they occur in wild-type larvae in around 1/1000 VNCs, thus they are experimentally challenging to detect [2^••^]. Most likely, these divisions are homeostatic, taking place as required. By contrast, genetic neuronal ablation in the embryonic VNC and stabbing and crush injury in larval VNCs, induce extensive Notch+Pros+NG proliferation [2••, 7••, 13]. Thus, the antagonistic functions of Notch and Pros endow NG with a mechanism that enables them to adjust their number during neural circuit formation, to maintain homeostasis, and to promptly divide on injury (Figure 1a,b).

Quiescent progenitors are also present in the adult brain, but went undetected for a long time [15•, 29•]. No proliferation can be observed with mitotic markers in the adult brain, but they can be detected, for example, with Mosaic Analysis with a Repressible Cell Marker (MARCM) clones as slow cycling progenitors [15•, 29•]. Apoptosis, stabbing injury and genetic neuronal ablation in the adult brain all induce cell proliferation [14•, 15•, 29•]. At least some of the adult progenitors are glia [14•, 15•], but whether all are glia, or if they are Notch+Pros+, like in the VNC, is unclear. Some of the progenitors for both neurons and glia in developing adult brain are Pros+Notch+, and Notch+ determines gliogenesis [30]. Either way, *Drosophila* neuropiles in VNC and brain retain quiescent or slow cycling progenitors throughout the life-course that regulate neuronal and glial cell number, enable homeostatic cell number adjustments, and are activated in response to injury.

A key driver of NG proliferation is *kon-tiki (kon)*, the *Drosophila* orthologue of mammalian *NG2*. *NG2* and *kon* encode transmembrane proteins and are highly evolutionarily conserved, with large extracellular domains, and smaller intracellular domains, both of which can be cleaved [31]. *kon* is dynamically expressed in NG during development [7^••^]: it is expressed in proliferating Notch+Pros+NG during axon guidance, is switched off as glial division ceases, and is switched on again in pupal and adult brain. Kon triggers proliferation of Notch+Pros+NG, but it cannot induce proliferation of Pros-negative NG. Kon is also required for the onset of glial differentiation in daughter cells, but glial differentiation maintenance depends on Pros [7^••^]. Pros regulates the expression of factors involved in neurotransmitter recycling, like Glutamine Synthetase and Ebony, and is required for axonal enwrapment [2••, 7••, 13, 22].

Upon CNS injury, the lesion typically first expands and then shrinks (Figure 1a) [2^••^]. The expansion coincides with extensive local cell death, and tissue shrinkage is associated with the glial regenerative response. Injury causes the up-regulation of *kon* expression in NG, and Kon induces proliferation of Notch+Pros+NG [7^••^] (Figure 1a,b). Following proliferation, Kon activates glial differentiation genes, including *pros* [7^••^]. This may also include genes involved in the repair response, such as *draper*, which encodes an engulfment receptor, as over-expression of *kon* greatly enhances repair [7^••^]. Kon is necessary and sufficient for the glial regenerative response to injury. If *kon* expression is knocked-down, the wound does not shrink and is heavily vacuolated; when *kon* is over-expressed, the wound shrinks further than in controls, and vacuolization is reduced [7^••^]. The injury-induced up-regulation of *kon* expression depends on Notch. Following cell division, Kon represses Notch, thus limiting the lifetime of *kon* expression to a narrow time window [7^••^]. Thus, the Notch-Kon loop enables glial proliferation, whilst setting a timer for the regenerative response (Figure 1b).

The injury-induced up-regulation of *kon* might also depend on NFκB homologue Dorsal, [2••, 7••]. NFκB normally rests inactive in the cytoplasm, but injury causes the TNF-dependent nuclear translocation of NFκB Dorsal in Pros+Notch+NG [2^••^]. Here, NFκB regulates gene expression and can activate cell proliferation. One of the targets of NFκB/Dorsal is *pros* [2^••^], which is activated in daughter cells enabling glial differentiation. In turn, Pros activates NFκB/Dorsal in daughter cells too, restoring the levels of NFκB protein in the cytoplasm, where it resides in the inactive state. NFκB/Dorsal is only activated again in response to injury. In this way, Pros prepares glia to respond to subsequent injuries [2^••^]. Thus, the Pros-NFκB loop primes glia to respond to injury (Figure 1a,b).

As well as restoring glial cell number, the regenerative response also enables glial differentiation (Figure 1a,b). The onset of glial differentiation depends on Kon, as *kon* activates *pros* expression, and loss of *kon* in development results in loss of the glial cell markers Repo, Ebony, GS, Naz and Pros [7^••^]. However, maintenance of the differentiated glial state does not depend on Kon, but on Pros instead [2••, 7••, 13]. After regenerative proliferation, Pros also represses *kon* expression [7^••^]. Thus, the Kon-Pros loop enables glial differentiation and cell number homeostasis. The sequential switch-off of *Notch* by Kon, and of *kon* by Pros, terminates the response to injury (Figure 1b).

This mechanism has two fundamental components: positive feedback loops (Pros-Notch and Pros-NFκB/Dorsal) that create ‘*go signals*’, driving a fast regenerative response to injury with nuclear translocation of NFκB/Dorsal, and a surge in Kon protein levels, possibly also Notch, together triggering glial proliferation; and negative feedback loops (Notch-Kon and Kon-Pros) that deliver ‘*stop signals*’ that switch off *Notch* and *kon* and activate *pros*, to repress proliferation and consolidate differentiation [2••, 7••] (Figure 1b). If the relative contributions of these genes are changed experimentally, the response to injury can be shifted from promotion to prevention of the regenerative response, or induction of tumourous over-growth [2••, 7••].

To conclude, the glial regenerative response is both plastic and homeostatic. The glial regenerative response is the re-activation of a developmental programme that coordinates glial proliferation with neural circuits, and maintains structural homeostasis throughout the life-course. This explains why such a mechanism would have been evolutionarily conserved. Importantly, it means that understanding developmental mechanisms is key to promoting regeneration and repair.

## Conserved mechanisms for NG-2 glia proliferation and differentiation

As in *Drosophila*, the mammalian spinal cord and brain are also populated with glial progenitors, NG2-glia that can divide (Figure 1c). Similar to flies, injury induces cell death and lesion expansion, followed by the compensatory proliferation of remaining NG2-glia and lesion shrinkage (Figure 1c). In mammals, *NG2* is expressed in OPCs, pericytes and microglia, but not in astrocytes, OLs or neurons [25^•^]. Similarly to *Drosophila* NG, NG2+OPCs have active Notch1 signaling, which maintains their proliferative state and inhibits their differentiation into OLs [32]. NG2 is required for OPC proliferation in development and upon injury (Figure 1c). *NG2*−/− knock-out mice have reduced OPC proliferation and fewer OLs [33^••^]. NG2 is also involved in the glial regenerative response, although results differ with the injury method. Upon cuprizone-induced demyelination, loss of *NG2* expression in knock-out mice does not affect proliferation or differentiation of OPCs [34]. However, loss of NG2 function exacerbates the damage caused by traumatic brain injury [35]. And lysolecithin-induced de-myelination in *NG2*−/− knock-out mice leads to reduced proliferation of OPCs [36, 37••]; and with conditional *NG2* knock-out either in OPCs or in myeloid cells, it reduces OPC proliferation, neuronal loss and cell debris clearance [37^••^]. In essence, the disparities reflect that NG2 is also required for the inflammatory response, and the commonalities that, much like in fruit-flies, NG2 is required for OPC proliferation in development and upon injury.

NG2 is not only expressed in OPCs but also in stem cells in adipose tissue and muscle, in pericytes that line the blood vessels and phagocytic macrophages/microglia [38, 39]. Like *NG2*, *kon* in *Drosophila* is also expressed in cells of the circulatory system (the dorsal vessel or heart), in muscles and in phagocytic glia (*Drosophila* NG are phagocytic) [40]. Such shared profiles indicate deep evolutionary conservation and relevant functions. *NG2* and *kon* and their interacting partners could reveal novel mechanisms for cell proliferation, phagocytosis and cell fate that could operate in multiple contexts.

In mammals, whilst injury readily induces NG2 glia proliferation, the differentiation of daughter cells into myelinating OLs does not always proceed successfully [1, 3]. The sustained up-regulation of Notch is one cause for OL differentiation failure [32], and a key challenge is to identify genes that antagonize Notch, and promote OL differentiation. *Drosophila pros* and its mammalian orthologue *prox1* encode homeo-domain transcription factors with a universal function in repressing cell proliferation and activating cell differentiation. *pros* orthologues are expressed and required by glia in flies, worms and mammals [2••, 13, 27, 41••, 42••]. In the mouse, *prox1* is not expressed in astrocytes, it is expressed in some OPCs at low levels, and at high levels in all OLs [25•, 26•]. Thus, either there are two types of NG2+OPCs (Pros+ and Prox1−), or Prox1 levels rise gradually as OPCs differentiate into OLs [26^•^]. The co-existence of NG2, Notch and Prox1 in OPCs mirrors the colocalisation of Notch, Pros and Kon in *Drosophila* NG. Conditional *prox-1* knock-out in the NG2+OPC cell lineage reduces OL number, prevents OL differentiation and increases NG2 cells and NG2 proliferation [26^•^]. Thus, like in *Drosophila*, Prox1 is required to promote OL differentiation in the mouse [26^•^]. This strongly indicates that *prox1* is a key gene to target in NG2 glia to promote the transition from OPCs to OLs, and sustain OL differentiation.

## Conclusion

To conclude, evolutionarily conserved molecular mechanisms regulate regenerative glial proliferation and differentiation in the fruit-fly VNC and mammalian spinal cord. Next it will be important to find out how general this molecular mechanism is - for instance, whether it functions also in injury responses in the brain - and why glial regeneration has such a remarkable effect on neuronal recovery. Intriguingly, glial Pros-1 regulates non-autonomously neuronal shape and function in *C. elegans* [41••, 42••]. Both fruit-flies and mammals bear slow cycling brain progenitors, but findings are either limited (fruit-flies) or controversial (mammals). For instance, in mammals whether progenitors are astrocytes or NG2 glia, and may be able to produce multiple CNS glial cell types and/or neurons, is highly debated [3, 43, 44]. Some striking similarities between fruit-fly and mammalian progenitors [11•, 14•, 15•, 45•] invoke further investigation. The manipulation of stem cells for transplantations and in vivo reprogramming to restore function following CNS damage [20] will continue to benefit from the discovery of molecular mechanisms through *Drosophila* genetics.

## Conflict of interest statement

Nothing declared.

## References and recommended reading

Papers of particular interest, published within the period of review, have been highlighted as:

• of special interest
•• of outstanding interest

## References

[1] Franklin R.J., Ffrench-Constant C. (2008). Remyelination in the CNS: from biology to therapy. Nat Rev Neurosci.

[2••] Kato K., Forero M.G., Fenton J.C., Hidalgo A. (2011). The glial regenerative response to central nervous system injury is enabled by pros-notch and pros-NFkappaB feedback. PLoS Biol.

[3] Dimou L., Gotz M. (2014). Glial cells as progenitors and stem cells: new roles in the healthy and diseased brain. Physiol Rev.

[4] Doherty J., Logan M.A., Tasdemir O.E., Freeman M.R. (2009). Ensheathing glia function as phagocytes in the adult Drosophila brain. J Neurosci.

[5] Neves J., Zhu J., Sousa-Victor P., Konjikusic M., Riley R., Chew S., Qi Y., Jasper H., Lamba D.A. (2016). Immune modulation by MANF promotes tissue repair and regenerative success in the retina. Science.

[6] He Z., Jin Y. (2016). Intrinsic control of axon regeneration. Neuron.

[7••] Losada-Perez M., Harrison N., Hidalgo A. (2016). Molecular mechanism of central nervous system repair by the Drosophila NG2 homologue kon-tiki. J Cell Biol.

[8] Hui S.P., Nag T.C., Ghosh S. (2015). Characterization of proliferating neural progenitors after spinal cord injury in adult zebrafish. PLoS One.

[9] McMurran C.E., Jones C.A., Fitzgerald D.C., Franklin R.J. (2016). CNS remyelination and the innate immune system. Front Cell Dev Biol.

[10] Crawford A.H., Chambers C., Franklin R.J. (2013). Remyelination: the true regeneration of the central nervous system. J Comp Pathol.

[11•] Petrenko V., Mihhailova J., Salmon P., Kiss J.Z. (2015). Apoptotic neurons induce proliferative responses of progenitor cells in the postnatal neocortex. Exp Neurol.

[12] Smith P.J., Howes E.A., Treherne J.E. (1987). Mechanisms of glial regeneration in an insect central nervous system. J Exp Biol.

[13] Griffiths R.L., Hidalgo A. (2004). Prospero maintains the mitotic potential of glial precursors enabling them to respond to neurons. EMBO J.

[14•] Kato K., Awasaki T., Ito K. (2009). Neuronal programmed cell death induces glial cell division in the adult Drosophila brain. Development.

[15•] Foo L.C., Song S., Cohen S.M. (2017). miR-31 mutants reveal continuous glial homeostasis in the adult Drosophila brain. EMBO J.

[16] Flygt J., Gumucio A., Ingelsson M., Skoglund K., Holm J., Alafuzoff I., Marklund N. (2016). Human traumatic brain injury results in oligodendrocyte death and increases the number of oligodendrocyte progenitor cells. J Neuropathol Exp Neurol.

[17] Emery B. (2010). Regulation of oligodendrocyte differentiation and myelination. Science.

[18] Sharp J., Frame J., Siegenthaler M., Nistor G., Keirstead H.S. (2010). Human embryonic stem cell-derived oligodendrocyte progenitor cell transplants improve recovery after cervical spinal cord injury. Stem Cells.

[19] Schneider S., Gruart A., Grade S., Zhang Y., Kroger S., Kirchhoff F., Eichele G., Delgado Garcia J.M., Dimou L. (2016). Decrease in newly generated oligodendrocytes leads to motor dysfunctions and changed myelin structures that can be rescued by transplanted cells. Glia.

[20] Amamoto R., Arlotta P. (2014). Development-inspired reprogramming of the mammalian central nervous system. Science.

[21] Awasaki T., Lai S.L., Ito K., Lee T. (2008). Organization and postembryonic development of glial cells in the adult central brain of Drosophila. J Neurosci.

[22] Peco E., Davla S., Camp D., Stacey S.M., Landgraf M., van Meyel D.J. (2016). Drosophila astrocytes cover specific territories of the CNS neuropil and are instructed to differentiate by Prospero, a key effector of Notch. Development.

[23] Omoto J.J., Lovick J.K., Hartenstein V. (2016). Origins of glial cell populations in the insect nervous system. Curr Opin Insect Sci.

[24] Stork T., Sheehan A., Tasdemir-Yilmaz O.E., Freeman M.R. (2014). Neuron-glia interactions through the Heartless FGF receptor signaling pathway mediate morphogenesis of Drosophila astrocytes. Neuron.

[25•] Cahoy J.D., Emery B., Kaushal A., Foo L.C., Zamanian J.L., Christopherson K.S., Xing Y., Lubischer J.L., Krieg P.A., Krupenko S.A. (2008). A transcriptome database for astrocytes, neurons, and oligodendrocytes: a new resource for understanding brain development and function. J Neurosci.

[26•] Kato K., Konno D., Berry M., Matsuzaki F., Logan A., Hidalgo A. (2015). Prox1 inhibits proliferation and is required for differentiation of the oligodendrocyte cell lineage in the mouse. PLOS ONE.

[27] Griffiths R.C., Benito-Sipos J., Fenton J.C., Torroja L., Hidalgo A. (2007). Two distinct mechanisms segregate Prospero in the longitudinal glia underlying the timing of interactions with axons. Neuron Glia Biol.

[28] Thomas G.B., van Meyel D.J. (2007). The glycosyltransferase Fringe promotes Delta-Notch signaling between neurons and glia, and is required for subtype-specific glial gene expression. Development.

[29•] Fernandez-Hernandez I., Rhiner C., Moreno E. (2013). Adult neurogenesis in Drosophila. Cell Rep.

[30] Chen Z., Del Valle Rodriguez A., Li X., Erclik T., Fernandes V.M., Desplan C. (2016). A unique class of neural progenitors in the drosophila optic lobe generates both migrating neurons and glia. Cell Rep.

[31] Trotter J., Karram K., Nishiyama A. (2010). NG2 cells: properties, progeny and origin. Brain Res Rev.

[32] Wang S., Sdrulla A.D., diSibio G., Bush G., Nofziger D., Hicks C., Weinmaster G., Barres B.A. (1998). Notch receptor activation inhibits oligodendrocyte differentiation. Neuron.

[33••] Kucharova K., Stallcup W.B. (2010). The NG2 proteoglycan promotes oligodendrocyte progenitor proliferation and developmental myelination. Neuroscience.

[34] Albrecht S., Hagemeier K., Ehrlich M., Kemming C., Trotter J., Kuhlmann T. (2016). Recovery from toxic-induced demyelination does not require the NG2 proteoglycan. PLOS ONE.

[35] Huang C., Sakry D., Menzel L., Dangel L., Sebastiani A., Kramer T., Karram K., Engelhard K., Trotter J., Schafer M.K. (2016). Lack of NG2 exacerbates neurological outcome and modulates glial responses after traumatic brain injury. Glia.

[36] Kucharova K., Chang Y., Boor A., Yong V.W., Stallcup W.B. (2011). Reduced inflammation accompanies diminished myelin damage and repair in the NG2 null mouse spinal cord. J Neuroinflamm.

[37••] Kucharova K., Stallcup W.B. (2015). NG2-proteoglycan-dependent contributions of oligodendrocyte progenitors and myeloid cells to myelin damage and repair. J Neuroinflamm.

[38] Birbrair A., Zhang T., Wang Z.M., Messi M.L., Enikolopov G.N., Mintz A., Delbono O. (2013). Skeletal muscle neural progenitor cells exhibit properties of NG2-glia. Exp Cell Res.

[39] Desiderio V., De Francesco F., Schiraldi C., De Rosa A., La Gatta A., Paino F., d’Aquino R., Ferraro G.A., Tirino V., Papaccio G. (2013). Human Ng2+ adipose stem cells loaded in vivo on a new crosslinked hyaluronic acid-Lys scaffold fabricate a skeletal muscle tissue. J Cell Physiol.

[40] Schnorrer F., Kalchhauser I., Dickson B.J. (2007). The transmembrane protein Kon-tiki couples to Dgrip to mediate myotube targeting in Drosophila. Dev Cell.

[41••] Kage-Nakadai E., Ohta A., Ujisawa T., Sun S., Nishikawa Y., Kuhara A., Mitani S. (2016). Caenorhabditis elegans homologue of Prox1/Prospero is expressed in the glia and is required for sensory behavior and cold tolerance. Genes Cells.

[42••] Wallace S.W., Singhvi A., Liang Y., Lu Y., Shaham S. (2016). PROS-1/Prospero is a major regulator of the glia-specific secretome controlling sensory-neuron shape and function in *C. elegans*. Cell Rep.

[43] Torper O., Ottosson D.R., Pereira M., Lau S., Cardoso T., Grealish S., Parmar M. (2015). In vivo reprogramming of striatal NG2 glia into functional neurons that integrate into local host circuitry. Cell Rep.

[44] Silva-Vargas V., Crouch E.E., Doetsch F. (2013). Adult neural stem cells and their niche: a dynamic duo during homeostasis, regeneration, and aging. Curr Opin Neurobiol.

[45•] Cheng L.C., Pastrana E., Tavazoie M., Doetsch F. (2009). miR-124 regulates adult neurogenesis in the subventricular zone stem cell niche. Nat Neurosci.

